# Role of Metabolism in Bone Development and Homeostasis

**DOI:** 10.3390/ijms21238992

**Published:** 2020-11-26

**Authors:** Akiko Suzuki, Mina Minamide, Chihiro Iwaya, Kenichi Ogata, Junichi Iwata

**Affiliations:** 1 Department of Diagnostic & Biomedical Sciences, School of Dentistry, The University of Texas Health Science Center at Houston, Houston, TX 77054, USA; akikosuz925@gmail.com (A.S.); mina.pe120@gmail.com (M.M.); Chihiro.Iwaya@uth.tmc.edu (C.I.); k.ogata@dent.kyushu-u.ac.jp (K.O.); 2 Center for Craniofacial Research, The University of Texas Health Science Center at Houston, Houston, TX 77054, USA; 3 MD Anderson Cancer Center UTHealth Graduate School of Biomedical Sciences, Houston, TX 77030, USA

**Keywords:** bone, metabolism, bone formation, bone homeostasis

## Abstract

Carbohydrates, fats, and proteins are the underlying energy sources for animals and are catabolized through specific biochemical cascades involving numerous enzymes. The catabolites and metabolites in these metabolic pathways are crucial for many cellular functions; therefore, an imbalance and/or dysregulation of these pathways causes cellular dysfunction, resulting in various metabolic diseases. Bone, a highly mineralized organ that serves as a skeleton of the body, undergoes continuous active turnover, which is required for the maintenance of healthy bony components through the deposition and resorption of bone matrix and minerals. This highly coordinated event is regulated throughout life by bone cells such as osteoblasts, osteoclasts, and osteocytes, and requires synchronized activities from different metabolic pathways. Here, we aim to provide a comprehensive review of the cellular metabolism involved in bone development and homeostasis, as revealed by mouse genetic studies.

## 1. Introduction

Bone (re)modeling is responsible for the growth and repair/regeneration of the tissue by maintaining a balance between bone matrix deposition and resorption during development and homeostasis [1]. Osteoblasts and osteoclasts are the cells responsible for bone deposition/mineralization and resorption, respectively [1]. The functions of bones include acting as a locomotorium coordinating with muscles, tendons, and joints, the support of posture, the protection of organs and brain, the storage of minerals, and hematopoiesis in the bone marrow. Bone has various metabolic networks and is well controlled to maintain homeostasis. The metabolic status of these cells affects bone formation and homeostasis via various biological reactions; in fact, compromised homeostasis and/or metabolic processes lead to various congenital skeletal disorders and bone diseases [1]. Metabolism as a whole comprises complex physical and biochemical processes that allow organisms to generate, maintain, and regenerate their structures and respond to environmental cues [2,3]. It involves numerous biochemical enzymatic reactions [2] necessary to sustain life, which are divided into three main metabolic pathways: (1) anabolism (the synthesis of complex macromolecules from the polymerization of simple molecules); (2) catabolism (the release of energy by the degradation of these molecules); and (3) degradation (the elimination of toxic substrates) [3]. A metabolic pathway is thus a series of enzymatic cascades that convert a molecule into another biochemical compound and are essential for homeostasis in organisms. Metabolic pathways can be categorized into anabolic and catabolic pathways. The anabolic pathway synthesizes complex molecules from simple or smaller molecules by consuming energy, whereas the catabolic pathway degrades complex molecules into simple or smaller molecules by releasing energy. Therefore, these pathways complement each other in terms of energy utilization, and the products and metabolites generated in one or more metabolic pathways may be used in other pathways as substrates or stored for future use. In addition to these two major pathways, the amphibolic pathway (e.g., citric acid cycle and Embden–Meyerhof pathway) is also involved in both the anabolic and catabolic metabolism [4].

An increasing number of studies show that type I and type II diabetes mellitus, obesity, and dyslipidemia are associated with risk of osteoporosis and a delay of bone healing [5,6,7,8,9]. For example, serum levels of high-density lipoprotein cholesterol (HDL-C) and triglycerides (TGs) are positively associated with bone mineral density (BMD) [10,11]. Individuals with hypercholesterolemia show decreased BMD [12]. In addition, patients with type II diabetes with hyperglycemia have a higher risk of failure of dental implants due to failure in osteointegration [13]. Moreover, the frequency of hip fractures is significantly associated with duration of type II diabetes [14]. Mice with type I diabetes induced by streptozotocin display impaired bone regeneration in the injured bones, and treatment with insulin, vitamin D, or both, can normalize it [15]. This line of evidence strongly suggests that abnormal energy catabolism influences bone formation and homeostasis. In this review, we will describe how catabolic pathways affect bone development and homeostasis (Table 1 and Table 2). 

## 2. Cholesterol Metabolism

Cholesterol is crucial as a source of numerous biomolecules, including bile acids, steroid hormones, and oxysterols, and is a vital component of cellular membranes; therefore, dysregulation of cholesterol synthesis is associated with various disorders and diseases [16]. It is also known that cholesterol modification of Hedgehog ligands–Sonic Hedgehog (Shh), Indian Hedgehog (Ihh), and Desert Hedgehog (Dhh–and receptor Smoothened (SMO) is crucial for their biological functions [17,18,19]. Hedgehog ligands are morphogens that play crucial roles in embryonic development and skeletogenesis [20,21,22]. Absence of a cholesterol moiety in SHH (ShhN) leads to a shorter distribution and lower activation of SHH signaling in limb buds, while it shows no difference in its biological functions compared to the molecules modified with cholesterol [23,24]. Mice overexpressing *ShhN* (*Sox2-Cre;Shh*^+*/*+^*;ShhN* or *Sox2-Cre;Shh^F/F^;ShhN*) exhibit polydactyly, holoprosencephaly, and cleft palate due to the long-range spread and activation of Shh signaling [24,25]. Thus, cholesterol modification in Hedgehog ligands is crucial for the regulation of Hedgehog signaling for morphogenesis. Moreover, the plasma membrane contains a large amount of lipids, especially cholesterol, which is essential for structural integrity, membrane fluidity, and membrane stability [26,27]. Cholesterol-rich micro-domains, lipid rafts, and caveolae act as a signaling center by assembling a variety of receptors and channels [28,29]. Thus, it is important to precisely control cholesterol synthesis and transportation through the regulation of cell viability and functions under physiological and pathological conditions. 

Cholesterol biosynthesis is regulated by a highly complex process that involves more than 30 reactions regulated by more than 15 enzymes [30]. De novo cholesterol biosynthesis (a.k.a. mevalonate pathway) begins with the generation of 3-hydroxy-3-methylglutaryl-coenzyme A (HMG-CoA) from acetyl-CoA. HMG-CoA reductase (HMGCR) then converts HMG-CoA into mevalonate at the ER membrane for the control of cholesterol synthesis through negative cholesterol feedback. Thus, the inhibition of HMGCR results in the suppression of cholesterol synthesis and a reduction in mature cholesterol levels. In zebrafish, inactivation of *hmgcr* by a genetic approach, or by treatment with a statin (an HMGCR inhibitor), results in shorter or lack of cartilage in the viscerocranium due to defects in condensation and the reduced survival of cranial neural crest cells during craniofacial development [31]. Statins stimulate bone formation in cultured osteoblasts [32,33,34,35] as well as in animal models [36,37,38]; more importantly, statins can rescue the skeletal dysplasia observed in mice with a gain-of-function mutation in the fibroblast growth factor receptor type III gene (*Fgfr3*) [39]. However, the clinical evidence of the effects of statins on bone is still controversial [40,41,42,43,44,45,46,47]. 

Farnesyl diphosphate synthase (FDPS, a.k.a. FPPS) converts isopentenyl pyrophosphate and dimethylallyl pyrophosphate into farnesyl pyrophosphate (FPP; a.k.a. FDP) and is known to be a target of nitrogen-containing bisphosphonates (N-BPs) [48,49]. FPP is further converted into squalene and geranylgeranyl diphosphate (GGPP) by farnesyl-diphosphate farnesyltransferase (FDFT1; a.k.a. SQS) and geranylgeranyl pyrophosphate synthase (GGPPS1), respectively. GGPP plays an important role in the activation of osteoclasts in bone formation and resorption [50,51]. A recent study shows that the inhibition of the mevalonate pathway with statins and N-BPs suppresses osteoclastogenesis and bone resorption via reduced binding of estrogen-related receptor alpha (ERRα), a nuclear receptor, to the promoter regions of its target genes [52]. In fact, long-term treatment with bisphosphonates causes bisphosphonate-related osteonecrosis of the jaw, a known side effect of these drugs [53,54]. Protein isoprenylation (farnesylation or geranylgeranylation) is one of the post-translational modifications and required for the activation of molecules. Farnesyltransferase catalyzes the conversion from farnesyl pyrophosphate to farnesyl proteins in farnesylation; geranylgeranyl transferase catalyzes the conversion from geranylgeranyl diphosphate to geranylgeranyl proteins in geranylgeranylation. The inhibition of farnesylation suppresses osteogenic differentiation and mineralization in human mesenchymal stem cells, while inhibition of geranylgeranylation accelerates osteogenic differentiation and mineralization [55]. In addition, inhibition of geranylgeranylation suppresses osteoclast differentiation, induces apoptosis in osteoclasts, and suppresses bone resorption [56]. Thus, protein prenylation plays an important role in the regulation of both osteoblastogenesis and osteoclastogenesis. 

**Figure 1:**
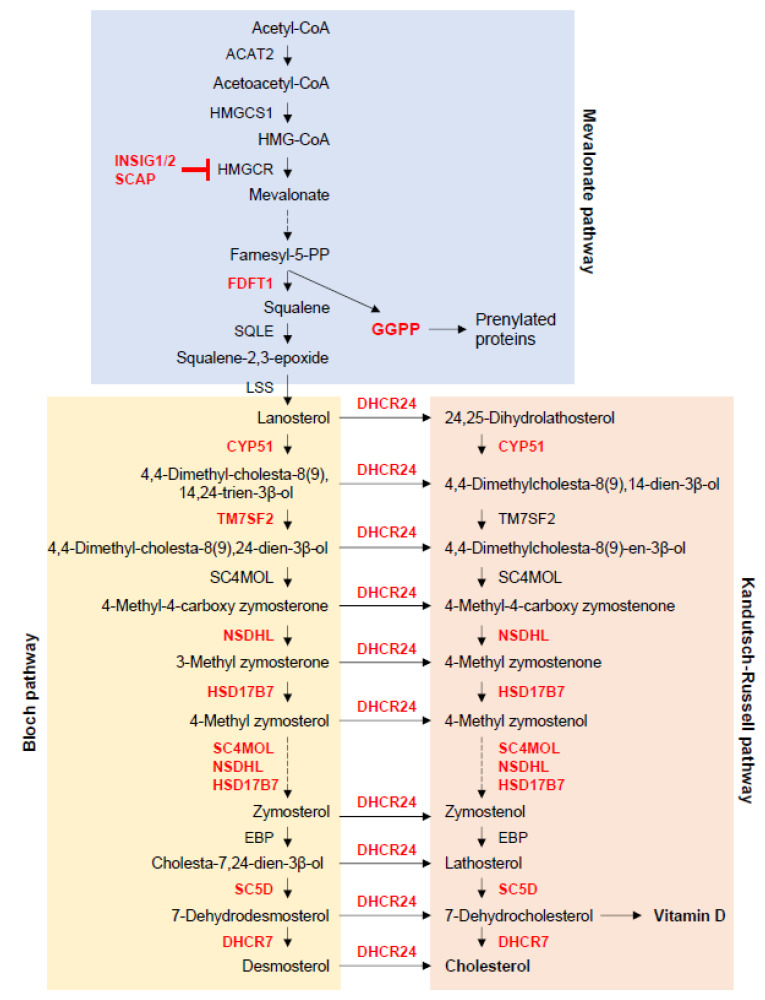
Cholesterol metabolism. This figure was drawn based on steroid biosynthesis pathway maps from the KEGG website. ACAT2, acetyl-CoA acetyltransferase 2; CYP51, cytochrome P450 family 51 subfamily; DHCR7, 7-dehydrocholesterol reductase; DHCR24, 24-dehydrocholesterol reductase; FDFT1, farnesyl-diphosphate farnesyltransferase 1; GGPP, geranylgeranyl pyrophosphate; HMGCR, HMG-CoA reductase; HMGCS1, 3-hydroxy-3-methylglutaryl-CoA synthase 1; HSD17B7, hydroxysteroid 17-beta dehydrogenase 7; LSS, lanosterol synthase; NSDHL, NAD(P)-dependent steroid dehydrogenase-like; SC4MOL (MSMO1), methylsterol monooxygenase 1; SC5D, sterol-C5-desaturase; SQLE, squalene epoxidase; TM7SF2, transmembrane 7 superfamily member 2.

In the cholesterol metabolic pathway, mice deficient for *Fdft1*, cytochrome P450, family 51 (*Cyp51*), and transmembrane 7 superfamily member 2 (*Tm7sf2*; a.k.a. *Dhcr14*), NAD(P) dependent steroid dehydrogenase-like (*Nsdhl*), hydroxysteroid (17-beta) dehydrogenase 7 (*Hsd17b7*), methylsterol monooxygenase 1 (*Sc4mol*), sterol-C5-desaturase (*Sc5d*), 7-dehydrocholesterol reductase (*Dhcr7*), 24-dehydrocholesterol reductase (*Dhcr24*), and insulin-induced gene 1 and 2 (*Insig1/2*) display defects in bone formation and/or homeostasis, as described below (Figure 1).

Squalene synthase *FDFT1* catalyzes the dimerization of FPP into squalene in the mevalonate pathway. Interestingly, treatment with zaragozic acid, an FDFT1 inhibitor, or excessive FPP in osteoblasts inhibits osteoblast differentiation and mineralization [57]. The homozygous *Fdft1* null mutation is embryonic lethal at embryonic day (E) 12.5, causing growth retardation and neurodevelopmental deficit [58]. Patients with squalene synthase deficiency, which is caused by autosomal recessive *FDFT1* mutations, exhibit facial dysmorphism, micrognathia, syndactyly, brain developmental defects, and developmental delay [59].

Additionally, lanosterol 14-α demethylase (CYP51A1 in humans and CYP51 in mice), a member of the cytochrome P450 superfamily, catalyzes the demethylation of lanosterol and 24,25-dihydrolanosterol in the cholesterol synthesis pathway via cytochrome P450 oxidoreductase (POR). *Cyp51* null mice (*Cyp51^−/−^* mice) exhibit a variety of skeletal defects, including cleft palate, micrognathia, brachycephaly, microglossia/aglossia, polydactyly/syndactyly, and malformation of long bones, as well as cardiac developmental defects, through altered sonic hedgehog and retinoic acid signaling [60].

TM7SF2 (a.k.a. DHCR14), a sterol delta (14)-D reductase, catalyzes the reduction of C14-unsaturated bond of lanosterol in a NADP-dependent manner during cholesterol biosynthesis. Mutations in *TM7SF2* cause Greenberg skeletal dysplasia (a.k.a. hydrops-ectopic calcification-moth-eaten or HEM skeletal dysplasia), an autosomal recessive chondrodystrophy characterized by fetal hydrops, short limbs, and abnormal chondro-osseous ossification. The bony defects in this syndrome include severe short-limbed dwarfism, postaxial polydactyly, and platyspondyly [61]. The Lamin B receptor (LBR), a paralog of TM7SF2, act bi-functionally as a lamin B receptor and delta14-reductase; TM7SF1 and LBR is functionally redundant for the delta14-reductase activity. Although mice with a deletion of *Tm7sf2* (*Tm7sf2*^∆*4-7*/∆*4-7*^) are almost comparable to wild-type mice, *Lbr*^+*/−*^; *Tm7sf2*^∆*4-7*/∆*4-7*^ mice exhibit shorter growth plates, less trabecular bones, growth retardation, cleft palate, and ataxia [62]. On the other hand, *Lbr^−/−^* mice exhibit disorganized hypertrophic chondrocytes (but normal growth plate length), immature trabecular bone, growth retardation, and ichthyosis [62,63]. 

NSDHL is a 3β-hydroxysterol dehydrogenase involved in the removal of two C4 methyl groups from 4,4-dimethylcholesta-8,24-dien-3β-ol, a sterol intermediate derived from lanosterol. Mutations in *NSDHL* cause X-linked dominant Congenital Hemidysplasia with Ichthyosiform nevus and Limb Defects (CHILD) syndrome, which is characterized by peculiar ichthyosiform nevus, hyperkeratotic plaques, and limb defects ranging from digital hypoplasia to complete amelia [64]. Heterozygous *Nsdhl* mutant female mice (bare patches [*Bpa*] mice) exhibit hyperkeratotic eruption and skeletal dysplasia with chondrodysplasia punctata [65]. 

SC5D converts lathosterol into 7-dehydrocholesterol, and *SC5D* mutations cause lathosterolosis, which is characterized by growth and mental retardation, short limbs, polydactyly/syndactyly, and craniofacial malformations, including cleft palate, micrognathia, midfacial hypoplasia, and calvarial defects [66]. Similarly, as seen in humans, mice with a deficiency for *Sc5d* (*Sc5d^−/−^* mice) exhibit cleft palate, micrognathia, agenesis of lower incisors, calvaria hypomineralization, malformation of long bones, and syndactyly/polydactyly [66,67].

The DHCR7 enzyme catalyzes the conversion of both 7-dehydrocholesterol (7-DHC) to cholesterol and 7-dehydrodesmosterol to desmosterol [68]. Mutations in *DHCR7* cause Smith-Lemli-Opitz syndrome (SLOS), which is characterized by cleft palate, postaxial polydactyly, 2-3 toe syndactyly, microcephaly, micrognathia, and mental retardation [69,70]. Mice deficient for *Dhcr7* (*Dhcr7^−/−^* mice) exhibit accelerated calvarial bone formation, cleft palate (in 9% of the mice), brain developmental defects, and immature lung development [71,72]. *Dhcr7* null osteoblasts show ciliogenesis defects (shorter and fewer primary cilia), leading to the upregulation of WNT/β-catenin signaling, which accelerates osteoblast differentiation and mineralization [72]. 7-DHC is also a precursor of vitamin D, a known regulator of calcium homeostasis, and is activated under ultraviolet B radiation (sunlight) in the skin.

DHCR24 catalyzes the reduction of the delta-24 double bond of sterol intermediates in cholesterol biosynthesis [73]. Autosomal recessive mutations in *DHCR24* cause desmosterolosis, which is characterized by cleft palate or high-arched palate, short limbs with osteosclerosis, contractures in the hands, and developmental brain defects [74,75,76]. *Dhcr24^−/−^* mice die before birth and present growth retardation and a wrinkleless taut skin, with no apparent defects in other parts of the body [77]. 

As described above, there are differences in the clinical features of the syndromes (e.g., lathostelosis, SLOS, and desmosterolosis) as well as in the phenotypes of *Sc5d^−/−^*, *Dhcr7^−/−^*, and *Dhcr24^−/−^* mice, suggesting that the accumulation of different cholesterol intermediates contributes to the pathogenesis of these diseases, rather than the absence of mature cholesterol [78]. 

Finally, INSIG1 and INSIG2 are ER-retention proteins that regulate cholesterol synthesis by modifying the activity of HMGCR and sterol regulatory element-binding protein (SREBP) cleavage-activating protein (SREBP cleavage activating protein; SCAP) in the ER membranes via a negative feedback mechanism triggered by high cholesterol status [79,80,81,82]. INSIG1/2 binds to HMGCR and promotes its degradation by the proteasome, resulting in decreased HMGCR enzymatic activity. On the other hand, INSIG1/2 binds to a complex of SCAP and SREBP, interrupting the translocation of the complex to the Golgi apparatus. At lower cholesterol levels, INSIG1/2 separate from the SCAP/SREBP complex, and this free complex is transported to the Golgi by coat protein complex II (COPII)-coated vesicles. SREBP is then cleaved at the Golgi membrane and translocated into the nucleus, thus inducing expression of its target genes [83]. *Insig1/2* double-null knockout (*Insig1^−/−^;Insig2^−/−^*) mice exhibit midline cleft face or cleft palate, micrognathia, exencephaly, and atelectatic lungs [84]. Moreover, mice with an *Insig1/2* deficiency in cranial neural crest cells (*Wnt1-Cre; Insig1^F/F^;Insig2^−/−^* mice), which form the majority of craniofacial structures, exhibit thin calvarial bone with low mineralization, resembling osteogenesis imperfecta [72]. Interestingly, *Insig1/2* mutant osteoblasts show abnormal multiple centrioles, supernumerary and longer primary cilia, resulting in compromised WNT/β-catenin signaling that leads to suppression of osteoblast differentiation and mineralization [72].

During chondrocyte differentiation, expressions of genes associated with cholesterol synthesis (e.g., *Hmgcr*, *Hmgcs1*, *Ldlr*, *Scap*, *Insig1*, *Srbp*) is downregulated [85]. The deletion of *Insig1/2* in either mesodermal cells or chondrocytes (*Prrx1-Cre; Insig1^F/F^; Insig2^−/−^* and *Col2a1-Cre; Insig1^F/F^; Insig2^−/−^* mice) results in a disorganized growth plate and chondrogenesis defects during endochondral ossification, which is manifested by short limbs and dwarfism [85]. *Prrx1-Cre; Insig1^F/F^; Insig2^−/−^* mice also exhibit a midline cleft, as seen in *Insig1^−/−^; Insig2^−/−^* mice. In addition, mice with a specific deletion of *Scap* in mesodermal cells or chondrocytes (*Prrx1-Cre; Scap^F/F^* and *Col2a1-Cre; Scap^F/F^* mice) show similar defects in growth plate organization and chondrogenesis, with short limbs and dwarfism [85]. The mesenchymal cells in *Prrx1-Cre; Scap^F/F^* mice show disorganized cellular condensation and suppressed proliferation and differentiation into chondrocytes. The suppression of cholesterol synthesis by a DHCR7 inhibitor AY9944 in 4 weeks-old rats suppresses Hedgehog signaling, leading to a reduction of chondrocyte proliferation and growth palate length [86]. Consistent with that observation, chondrocytes from *Col2a1-Cre; Scap^F/F^* and *Col2a1-Cre; Insig1^F/F^; Insig2^−/−^* mice show downregulated and upregulated Hedgehog signaling, respectively [85]. Interestingly, normalization of upregulated intracellular cholesterol synthesis in *Col2a1-CreERT2;Idh1^R132Q/R132Q^* mice by loss of *Scap* (*Col2a1-CreERT2;Idh1^R132Q/R132Q^;Scap^F/F^* mice), or by treatment with lovastatin, suppresses chondrogenic tumor growth [87]. These studies suggest that either the upregulation or downregulation of intracellular cholesterol synthesis compromises osteogenesis and chondrogenesis. In addition, there is a positive feedback loop for expression of genes regulating the sterol metabolic pathway (e.g., *Cyp11a1*, *Cyp39a1*, *Cyp51*, *Lss*, *Dhcr7*) regulated by *Runx2*. For instance, during osteogenic differentiation, *Cyp11a1*, which catalyzes the conversion from cholesterol to pregnolone, is a direct target of RUNX2 and suppresses cell proliferation in MC3T3-E1 osteoblast cells [88]. These results suggest that cholesterol synthesis is directly linked to osteogenic differentiation.

## 3. Fatty Acid Metabolism

**Figure 2:**
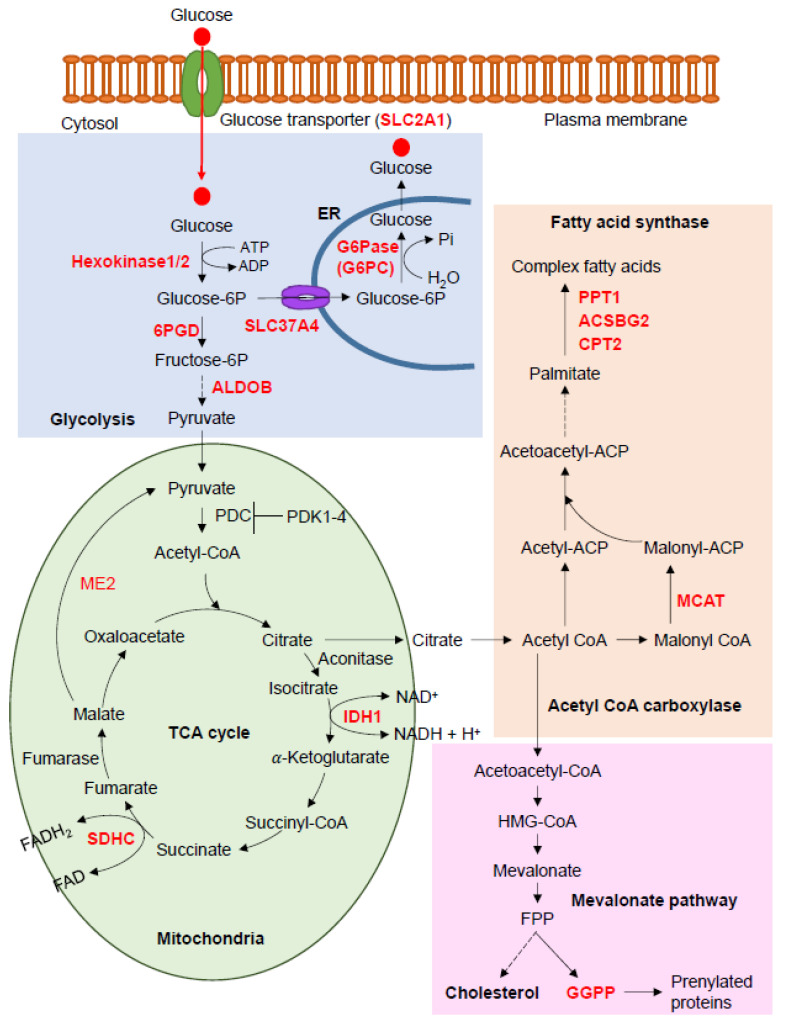
Fatty acid metabolism, glycolysis, and tricarboxylic acid cycle (TCA) cycle. Fatty acid biosynthesis, fatty acid elongation, glycolysis pathway, and TCA cycle were drawn based on maps from the KEGG website; 6PGD, 6-phosphogluconate dehydrogenase, decarboxylating; ACSBG2, acyl-CoA synthetase bubblegum family member 2; ALDOB, aldolase, fructose-bisphosphate B; CPT2, carnitine palmitoyltransferase 2; FPP, farnesyl pyrophosphate; G6Pase, glucose 6-phosphatase; IDH1, isocitrate dehydrogenase (NADP(+)) 1; MCAT, malonyl-CoA-acyl carrier protein transacylase; ME2, malic enzyme 2; PDC, pyruvate dehydrogenase complex; PDK1, pyruvate dehydrogenase kinase 1; PPT1, palmitoyl-protein thioesterase 1; SDHC, succinate dehydrogenase complex subunit C; SLC24A1, solute carrier family 24 member 1; SLC37A4, solute carrier family 37 member 4.

Fatty acid metabolism involves an enzymatic cascade that degrades fatty acids into bioactive substrates and synthesizes straight-chain fatty acids to be stored as triglycerides in adipose tissues. The catabolic pathway starts with the release of free fatty acids from glycerol, consumed in the diet or derived from triglycerides in adipose tissue through lipolysis, followed by transport to peripheral cells and the entire body, according to its needs. In the cytosol, long-chain fatty acids are catalyzed with ATP to acyl-CoA and further broken down to acetyl-CoA by β-oxidation to enter the tricarboxylic acid cycle (TCA) cycle in the mitochondrial matrix. On the other hand, acetyl-CoA is a substrate for straight-chain fatty acid synthesis. Triglycerides, phospholipids, precursors of eicosanoid hormones and second messengers, and ketone bodies are all produced in both the catabolic and synthetic pathways. A recent study suggests that single nucleotide polymorphisms (SNPs) in genes associated with fatty acid synthesis (*MCAT*, *PPT1*, *ACSL5*, *HSD17B12*, and *ACADL*) are associated with non-syndromic cleft lip with/without palate in humans [89]. A proteomic analysis demonstrated that expression of inflammation-related proteins and HSD17B12 is significantly elevated in chondrocytes isolated from articular cartilage of osteoarthritis patients [90]. These results suggest that fatty acid synthesis is closely associated with skeletal disorders. Indeed, mice with a deficiency of malonyl-CoA-acyl carrier protein transacylase (*Mcat*; inducible *Mcat*^−/−^ mice) [91], palmitoyl-protein thioesterase 1 (*Ppt1*; *Ppt1^−/−^* mice) [92]), acyl-CoA synthetase bubblegum family member 2 (*Acsbg2*; *Acsbg2^−/−^* mice), and carnitine palmitoyltransferase 2 (*Cpt2*; *Cpt*^+*/−*^ mice) display various defects in bone formation and/or homeostasis (Figure 2). 

MCAT is a crucial enzyme in fatty acid biosynthesis and is responsible for transferring the malonyl moieties from malonyl-CoA to mitochondrial acyl carrier proteins. Mice with a deficiency in *Mcat* in almost all tissues, due to tamoxifen-inducible *Cre* expression starting at 4−6 weeks of age (*Mcat^F/F^;Esr1-CreER* mice), exhibit kyphosis at 10 months without any unusual morphologic changes in bone [91].

PPT1 hydrolyzes long-chain fatty acyl-CoA during fatty acid synthesis, in addition to hydrolyzing fatty acids from modified cysteine residues of proteins that undergo lysosomal degradation. *Ppt1^−/−^* mice exhibit thick calvaria and neurological abnormalities [92] through either dysregulated fatty acid metabolism or disrupted lysosomal protein degradation. In humans, autosomal recessive mutations in *PPT1* cause infantile neuronal ceroid lipofuscinosis 1 with accumulation of fatty acyl cysteine thioesters in neural cells, which leads to neuronal cell death [93,94].

ACSBG2, a long-chain fatty acid-CoA ligase isozyme, converts free long-chain fatty acids to fatty acyl-CoA esters, and *Acsbg2^−/−^* mice exhibit low bone mineral density (reported by the International Mouse Phenotyping Consortium [IMPC] at https://www.mousephenotype.org). 

CPT2 oxidizes long-chain fatty acids by adding CoA at the mitochondrial inner membrane. The deletion of this enzyme in the fatty acid β-oxidation pathway in *Cpt2^−/−^* mice results in abnormal vertebrae morphology (reported by the IMPC); however, it remains unclear how these enzymes affect bone formation and homeostasis.

HSD17B12 (estradiol 17-β-dehydrogenase 12) is an enzyme that catalyzes the conversion of 3-ketoacyl-CoA to 3-hydroxyacyl-CoA in an NADP-dependent manner in fatty acid elongation. ACSL5 (acyl-CoA synthetase long chain family member 5) catalyzes the conversion of long-chain fatty acids to active fatty acyl-CoA esters, whereas ACADL (acyl-CoA dehydrogenase, long chain) catalyzes β-oxidation in the mitochondria to break down fatty acids into acetyl-CoA. However, mice with mutations in these genes have not yet been analyzed in detail. 

The uptake of long-chain fatty acids is made through fatty acid translocase CD36 [cluster of differentiation 36; a.k.a. fatty acid translocase (FAT)] [95]. *Cd36* null mice (*Cd36^−/−^*) show no defect at the initial stages of bone development; however, they show lower bone mass as adults due to the suppression of osteoblast differentiation [96]. Long-chain fatty acids mediate signaling through their binding to G-protein-coupled receptors, GPR40 and GPR120, in bone cells [97,98,99,100,101]. GPR120 is expressed at higher levels in mature osteoblasts and osteoclasts compared to precursor cells. The activation of the GPR120-mediated signaling pathway suppresses bone resorption through the inhibition of osteoclast differentiation induced by the RANKL‒NFκB‒NFATc1 pathway [100] and promotes osteogenesis via the Ras‒ERK1/2 signaling pathway [99]. Activation of the free fatty acid receptor G-coupled protein receptor 40 (GPR40)-mediated signaling pathway stimulates osteoblastogenesis, but inhibits mineralization [97], and suppresses osteoclastogenesis through the inhibition of the RANKL‒NFκB‒NFATc1 pathway [101]. *Gpr40* knockout (*Gpr40^−/−^*) mice exhibit an osteoporotic bone phenotype (e.g., low bone mass, low bone mineral density, low trabecular thickness/number) [101]. Treatment of ovariectomized mice with a GPR40 agonist protect from bone loss by normalizing osteoclast activity [101]. In addition, essential fatty acids (alpha-linolenic acid and linoleic acid) have been suggested to regulate calcification and bone resorption [102,103,104,105]. For instance, β-oxidation of fatty acids is required for bone formation and maintenance. Female mice with a deficiency for carnitine palmitoyltransferase-2 (*Cpt2*), which catabolizes compounds through β-oxidation, in osteoblasts (*Oc-Cre; Cpt2^F/F^*) exhibit decreased bone acquisition due to the suppression of osteoblast differentiation [106]. In addition, short-chain fatty acids are up-taken through GPR41 or GPR43 (a.k.a. FFAR2) and inhibit osteoclastogenesis and bone resorption under physiological and pathological (e.g., ovariectomized osteoporotic) conditions [107]. These results suggest that free fatty acids and their synthesis are associated with bone formation and bone loss in physiological and pathological conditions.

## 4. Glycolysis and Gluconeogenesis

Glycogen, a long, branched polymer of glucose residues, is a readily mobilized storage form of glucose that is mainly present in the liver and skeletal muscles; when the body needs energy, glycogen is broken down into glucose. In the liver, glycogen synthesis and degradation are regulated for the maintenance of blood glucose levels as required by the organism, but in muscles and other tissues these processes are regulated based on their specific needs. It is well known that patients with hyperglycemia or diabetes show decreased bone remodeling. Excessive glucose addition inhibits cell proliferation and osteogenic differentiation in a dose-dependent manner in human bone marrow mesenchymal stem cells and osteoblast cell lines MG63 and MC3T3-E1 [108,109,110]. Glucose is up-taken through glucose transporters SLC2A1-4 (solute carrier family 2, member 1-4; a.k.a. GLUT1-4). Among them, SLC2A1 is expressed in osteoclasts, and its expression is upregulated over time during osteoclast differentiation and maturation. In the growth plate, SLC2A1 is expressed only in upper hypertrophic chondrocytes [111,112]. Female mice with a deletion of *Slc2a1* in monocyte-derived osteoclasts (*Lys2-Cre;Slc2a1^F/F^*) show defects in osteoclastogenesis, but not males, leading to increased trabecular bone mass [113]. In addition, the expression of *Slc2a1* is upregulated over time during osteoblast differentiation and suppressed by tumor suppressor gene *p53* [114] and induced by *Runx2* [115]. Mice with a deletion of *Slc2a1* in mesoderm-derived cells (*Dermo1-Cre; Slc2a1^F/F^*), pre-osteoblasts (*Osx-Cre; Slc2a1^F/F^* and *Osx-CreER^T2^; Slc2a1^F/F^*), and osteoblasts (*Bglap-Cre; Slc2a1^F/F^*) also exhibit severe bone mineralization defects with reduced trabecular bone mass in both endochondral and intramembranous ossification [115,116]. Mice with a mesenchymal cell-specific deletion of *Slc2a1* in the limb bud and craniofacial region (*Prx1-Cre; Slc2a1^F/F^*) exhibit short long bones with a disorganized columnar structure in the proliferation and hypertrophic zones of the growth plate, the suppression of cartilage matrix synthesis, and modestly delayed ossification [111]. *Slc2a1* is also involved in stabilization of RUNX2 to promote osteoblast differentiation [115]. Thus, glucose uptake and consequent aerobic glycolysis play crucial roles in bone formation and remodeling.

Glycolysis is a biochemical cascade that is conserved in almost all organisms and that catalyzes glucose to pyruvic acid (a.k.a. pyruvate), nicotinamide adenine dinucleotide (NADH), ATP, and other intermediates, in the cytosol. In osteoblasts, 80% of the ATP is produced by aerobic glycolysis [117]. For instance, NAD^+^-dependent mitochondrial malic enzyme ME2, which catalyzes the conversion of malate to pyruvate in the mitochondria, is upregulated during osteoblast differentiation. The knockdown of *Me2* in osteoblasts suppresses glucose consumption and cell proliferation and differentiation [117]. In addition, a recent study showed that aerobic glycolysis is required for osteoclastogenesis and bone resorption [113]. In the glycolysis and gluconeogenesis pathway, mice with a deficiency in the glucose-6-phosphatase catalytic subunits (*G6pc*), i.e., 6-phosphogluconate dehydrogenase (*Pgd*), aldolase B (*Aldob*), display defects in bone formation and/or homeostasis (Figure 2). 

Glucose-6-phosphatase (G6Pase), a multi-subunit integral membrane protein of the endoplasmic reticulum (ER), is a crucial enzyme in gluconeogenesis and catalyzes the hydrolysis of D-glucose-6-phosphate (glucose-6P, G6P) to D-glucose and orthophosphate, thus generating a phosphate group and free glucose. G6Pase comprises a catalytic subunit and transporters for glucose-6P, phosphate (Pi), and glucose and is mainly expressed in the liver and kidneys for glucose homeostasis. Mutations in *G6PC*, one of the three genes encoding the G6Pase catalytic subunits in humans (*G6PC1*, *G6PC2*, and *G6PC3*), cause glycogen storage disease type Ia (GSD-Ia), which is characterized by neuropathy, hepatic adenocarcinoma, and osteopenia [118,119,120]. Mice with a loss of *G6pc* (*G6pc^−/−^*) exhibit cartilage dysplasia [121], delayed ossification in the growth plate of bones, decreased bone dimensions, and decreased levels of growth hormone (GH) and insulin-like GH, and hepatic GH signaling [122]. Insulin-like growth factor 1 (IGF1) is a peptide hormone; its production is stimulated by GH in the liver [123]. IGF1 signaling is mediated by binding to the IGF1 receptor type I (IGF1R). The GH‒IGF1 axis is a well-known regulator for bone growth and remodeling through the stimulation of cell proliferation, osteoblastogenesis and osteoblast activity, osteoblast-to-osteocyte transition, osteoclast activity, and chondrocyte differentiation at the growth plates [124,125,126,127]. *Igf1* null (*Igf1^−/−^*) mice exhibit decreased cell proliferation in the growth plate, delayed bone development, and growth retardation [128,129]. Mice with a deficiency for IGF1 receptor type I in pre-osteoblasts and pre-hypertrophic chondrocytes (*Osx-GFP: Cre; Igf1r^F/F^*) exhibit shorter hypertrophic zone in the growth plates and suppressed chondrocyte differentiation/maturation, resulting in the retardation of postnatal bone growth [130]. Mice with a deficiency for *Igf1r* in osteoblasts (*Col1a1-Cre; Igf1r^F/^*^F^) exhibit decreased post-fracture bone formation due to suppressed osteoblastogenesis [131]. Mice with a tamoxifen-induced deficiency of *Igf1r* in bone marrow mesenchymal cells (*Nestin-Cre^ER^; Igf1r^F/F^*, tamoxifen induction at 3 weeks of age) exhibit decreased trabecular bone mass due to suppression of osteoblast differentiation [132]. These results suggest that the reductions in serum GH and IGF1 are responsible for the impaired bone growth phenotype seen in *G6pc^−/−^* mice. SLC37A4 (G6P translocase; a.k.a. G6PT), working together with G6Pase, transports D-glucose 6-phosphate (glucose-6P) from the cytoplasm to the ER and is known to be a causative gene of GSD-Ib, which is characterized by osteopenia, also seen in patients with GSD-Ia caused by mutations in G6Pase [120]. Mice with a *Slc37a4* deficiency (*Slc37a4^−/−^* mice; a.k.a. *G6PT^−/−^* mice) exhibit seizures, suppressed hematopoiesis, and delayed bone development at 3 weeks of age but they catch up by 6 weeks of age [133]. These findings indicate that appropriate glycogen storage and degradation are essential for bone homeostasis. 

In the cytosol, G6P is converted to fructose-6 phosphate (F6P) by phosphogluconate dehydrogenase (6PGD; a.k.a. 6PGDH), which is further converted to pyruvate by aldolase B (a.k.a. fructose-bisphosphate B). Pyruvate is a key metabolite connecting glycolysis, the TCA cycle, cholesterol synthesis, lipid catabolism, and amino acid metabolism through acetyl-CoA. The pyruvate dehydrogenase complex (PDC), which includes three catalytic domains, catalyzes the conversion from pyruvate and NAD^+^ to acetyl-CoA, CO_2_, and NADH in the mitochondria. Mice with a deficiency of either *Pgd* (*Pgd*^+*/−*^ mice) or *Aldob* (*Aldob^−/−^* mice) exhibit low bone mineral content and density (reported by the IMPC). Patients with autosomal dominant *PGD* mutations are asymptomatic, whereas those with autosomal recessive *ALDOB* mutations show hereditary fructose intolerance, but no bone abnormalities. Future studies may identify the contribution of these genes in bone diseases. Pyruvate dehydrogenase complex activity is inactivated by pyruvate dehydrogenase kinase 1-4 (PDK1-4). While *Pdk4^−/−^* mice normally develop and maintain bone homeostasis, *Pdk4^−/−^* mice show reduced bone mass due to enhanced osteoclastogenesis and bone resorption under unloading conditions [134]. Bone marrow mesenchymal cells isolated from *Pdk4^−/−^* mice show suppression of osteoclastogenesis but no defect in osteoclast fusion. In recombination assays, osteoblasts isolated from *Pdk4^−/−^* mice failed to induce osteoclastogenesis of monocytes isolated from wild-type mice due to decreased *Rankl* expression [134]. Ovariectomy, a procedure that induces estrogen-dependent osteoporosis, of *Pdk2* null (*Pdk2^−/−^*) mice show reduced osteoclast number, suppressed osteoclastogenesis, and suppressed bone resorption through the RANKL‒CREB‒c-Fos‒NFATc1 pathway [135]. Thus, the suppression of transformation to acetyl-CoA causes bone loss. 

## 5. Glycogenolysis and Glycogenesis

Dietary carbohydrates are digested into glucose by amylase and dissolved into the bloodstream. At each organ, glucose is up-taken into the cytosol through glucose transporters and is synthesized to glycogen. Glycogen is a multi-branched polysaccharide containing multi-units of glucose and stored as an energy source, mainly in the liver and skeletal muscles [136]. Glycogenolysis initiates with the breakdown of glycogen into glucose 1-phosphate (G1P) by AGL and glycogen phosphorylase. G1P is further broken down into G6P by phosphoglucomutase. In the growth plate in young rats, glycogen is abundant in the resting, pre-hypertrophic, and hypertrophic chondrocytes but is less abundant in the proliferative and late-hypertrophic chondrocytes [137]. Inversely, glycogen degradation enzymes (glycogen phosphorylase and G6Pase) are highly active in the proliferative, pre-hypertrophic, and hypertrophic chondrocytes and are suppressed in the resting and late-hypertrophic chondrocytes [112,137]. G6Pase is expressed at higher levels in osteoblasts than in osteocytes and osteoclasts in the metaphyses of young rats [138]. On the other hand, glycogen synthesis (glycogenesis) starts with the conversion of glucose to G6P by glucokinase in the liver, or hexokinase 1 and -2 in other organs. G6P is then converted to G1P by phosphoglucomutase. UDP-glucose pyrophosphorylase 2 (UGP2) then catalyzes the transformation of G1P and UTP to UDP-glucose and pyrophosphate (PPi). Finally, UDP-glucose and glycogenin are converted to glycogen by UDP-glucose-glycogen glucosyltransferase (a.k.a. glycogen synthase) and branching enzymes.

Amylo-alpha-1, 6-glucosidase, 4-alpha-glucanotransferase (AGL), which is expressed in the liver and skeletal muscles, plays a role in glycogen degradation. AGL has two enzymatic activities—glucosyltransferase and glucosidase—and breaks down (debranches) glycogen into G1P, together with glycogen phosphorylase [139]. In humans, mutations in *AGL* are associated with glycogen storage disease type III (GSD-III), an autosomal recessive metabolic disorder caused by the accumulation of glycogen in the liver and skeletal muscles, resulting in organ dysfunction. GSD-III is further characterized into two subtypes: (1) GSD-IIIa, which affects only the liver; and (2) GSD-IIIb, which involves both the liver and skeletal muscles. Unlike patients with GSD-IIIa, patients with GSD-IIIb exhibit low bone mineral density [119,140,141,142]. In addition, *Agl* knockout mice (*Agl^−/−^* mice) exhibit kyphosis [143]. However, the exact mechanism of the skeletal dysplasia in *Agl^−/−^* mice and the contribution of glycogen metabolism to bone homeostasis will be further examined in mice with a specific deletion of *Agl* in bone cells.

**Figure 3:**
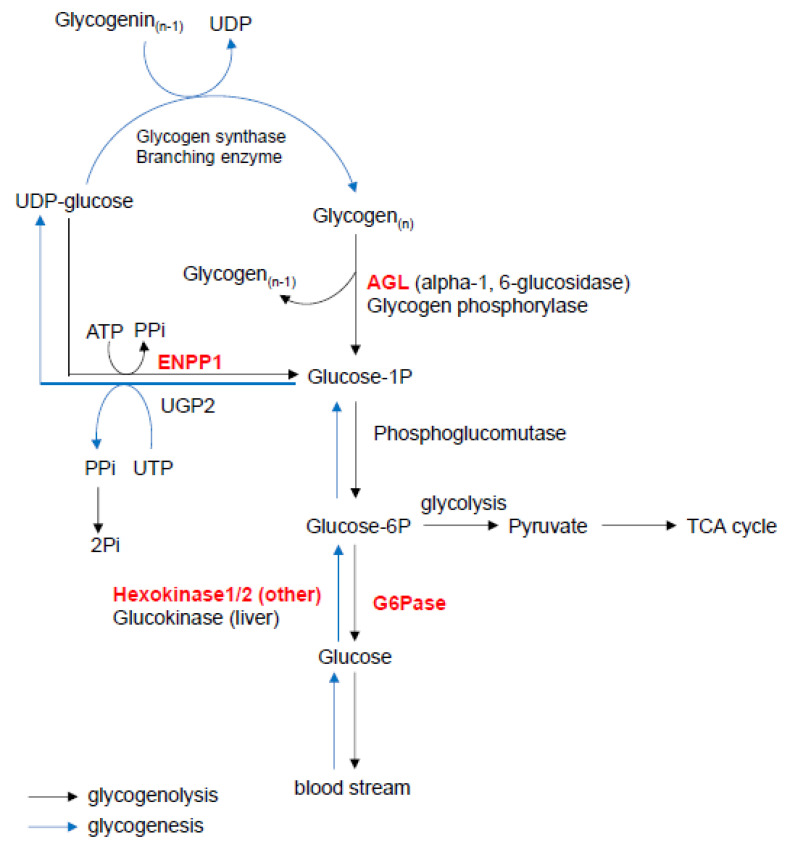
Glycogen degradation and glycogenesis. This figure was drawn based on the glycogen metabolism pathway map from the KEGG website. AGL, amylo-alpha-1,6-glucosidase 4-alpha-glucanotransferase; ENPP1, ectonucleotide pyrophosphatase/phosphodiesterase 1; G6Pase, glucose 6-phosphatase; PPi, pyrophosphate; Pi, phosphate; UDP, uridine diphosphate; UGP2, UDP-glucose pyrophosphorylase 2; UTP, uridine triphosphate.

In addition, enzyme ectonucleotide pyrophosphatase/phosphodiesterase 1 (ENPP1) plays a role in the regulation of pyrophosphate levels by generating inorganic pyrophosphate (PP_i_) from adenosine triphosphate (ATP), acting as a suppressor of calcification under various pathological conditions [144,145]. In glycogen degradation and glycogenesis, ENPP1 catalyzes the dephosphorylation of uridine diphosphate glucose (UDP-glucose) into G1P and PP_i_, which is an anti-mineralization factor under physiological conditions [146]. In humans, autosomal recessive *ENPP1* deficiency causes hypophosphatemic rickets type 2, which is characterized by low bone mass and osteopenia [147,148]. The loss of *Enpp1* results in abnormal bone mineralization in *twy* mutant mice, which have a spontaneous nonsense mutation in *Enpp1* (p.Gly568Ter), as well as in mice with a spontaneous *Enpp1* deletion, in mice with a chemically induced (by the mutagen N-ethyl-N-nitrosourea [ENU]) single point mutation (c.T737A), and *Enpp1^−/−^* null mice [148,149,150,151,152,153,154]. In addition, all of these *Enpp1* mutant mice exhibit excessive calcification of the articular cartilages/joints, ankylosis/joint fusion, exostosis, and calcification in blood vessels [148,150,155,156,157]. The accelerated calcification in *Enpp1*-deficient osteoblasts is caused by decreased extracellular PP_i_ levels and downregulated osteopontin expression [152]. However, because ATP, a complex organic chemical that participates in many biological processes, is one of the primary substrates for ENPP1 [158], the contribution of ENPP1 to glycogen degradation and glycogenesis needs to be evaluated in mice with a specific deletion of *Enpp1* in bone cells (Figure 3).

## 6. TCA Cycle

The TCA cycle (a.k.a. the citric acid cycle or the Krebs cycle) is an essential metabolic cycle present in the mitochondria of all aerobic organisms. Through glycolysis, pyruvic acid and fatty acyl-CoA are converted into acetyl-CoA, with subsequent synthesis of guanosine triphosphate (GTP)/ATP, NADH, and amino acids. Studies in mice have shown that a deficiency of *Idh1* (isocitrate dehydrogenase 1, cytosolic) and *Sdhc* (succinate dehydrogenase complex subunit C) in the TCA cycle pathway induces defects in bone formation and/or homeostasis (Figure 2). 

Isocitrate dehydrogenase 1 (IDH1) is an enzyme that catalyzes the oxidative decarboxylation of isocitrate and NADP^+^, producing α-ketoglutarate (a.k.a. 2-oxoglutarate) and NADPH. Mutations in *IDH1* cause several cancers such as solitary or multiple enchondromas, acute myeloid leukemia, and glioma [159]. Mutations in *IDH1* also accelerate the conversion of α-ketoglutarate to D-2-hydroxyglutarate and the accumulation of D-2-hydroxyglutarate, which is known as an oncometabolite competing with α-ketoglutarate in binding to histone and DNA demethylases, leading to chromatin hypermethylation [160,161]. The exogenous supplementation of D-2-hydroxyglutarate inhibits osteoblast differentiation from mesenchymal stem cells [162]. Zebrafish embryos treated with 2-hydroxyglutarate exhibit a failure of bone formation without affecting cartilage development [162]. In addition, an accumulating number of genetic studies suggests that IDH1 plays crucial roles in bone and cartilage formation. For example, mice with a knock-in mutation in the *Idh1* gene causing human enchondroma (*Col2a1-Cre; Idh1^R132Q/^*^+^ mice) exhibit growth plate disorganization and cartilaginous dysplasia [163]. Moreover, mice with the inducible mutation (*Col2a1-CreERT2; Idh1^R132Q/^*^+^) develop enchondroma-like cartilage around the growth plate [163]. Known oncogenic mutations in the *IDH1* gene in humans (such as R132C, R132Q, and R132H) upregulate enzymatic activity for the conversion of α-ketoglutarate to D-2-hydrozyglutarate, an inhibitor of histone demethylase [163,164,165]. In mice, the expression of hypertrophic chondrocyte markers *Runx2* and *Col10a1* and cell proliferation are upregulated at the proliferating zone of the growth plate in mice with oncogenic *Idh1* mutations, indicating that a proper amount of intermediates from the TCA cycle is vital for the development and homeostasis of cartilage and bone. 

Succinate dehydrogenase (SDH) participates in both the TCA cycle and the electron transport chain as the mitochondria respiratory chain complex II at the mitochondria’s inner membrane, which catalyzes the oxidation of succinate converting it to fumarate. Succinate dehydrogenase subunit C (SDHC) is one of the four subunits of SDH [166]. Mice heterozygous for the *Sdhc* null mutation (*Sdhc^+/−^* mice) exhibit decreased bone mineral content in females, but increased content in males without any tumorigenesis (reported by the IMPC). In humans, patients with autosomal dominant mutations in the *SDHC* gene show benign hereditary pheochromocytomas/paragangliomas type III without any bone abnormality. Future studies may identify the contribution of *SDHC* in bone diseases.

## 7. Phospholipid Metabolism

Phospholipids are synthesized in the ER and include a hydrophobic fatty acid tail and a hydrophilic head that form the lipid bilayer in the eukaryotic plasma membrane. The lipid bilayer comprises, in addition to phospholipids, several other molecules such as proteins, cholesterols, and glycoproteins that contribute to the membrane’s function and fluidity. The metabolites of phospholipids also serve as second messengers in signal transduction [e.g., phosphatidic acid (PA), phosphatidylinositol-(4,5)-bisphosphate (PIP2), diacylglycerol (DAG), and prostaglandins]. Phosphatidylserine receptors such as TIM4, BAI1, and STAB2 are expressed in mature osteoclasts. Blocking these receptors or reducing extracellular phosphatidylserine inhibits fusion of pre-osteoclasts without affecting ostoclastogenesis [167,168]. Interestingly, extracellular phosphatidylserine induces apoptosis in mature osteoclasts through TIM4 and BAI1 receptors [167]. Bone filling with hydroxyapatite containing phosphatidylserine-liposomes accelerates osteoblast differentiation and bone regeneration at injured sites of rat calvaria compared to hydroxyapatite alone [169]. Lysophosphatidic acid (LPA) is a phospholipid-derivative growth factor, which is converted from lysophosphatidylcholine that acts through G-protein-coupled receptors LPAR1-6. Treatment with lysophosphatidic acids enhances osteoblast differentiation, mineralization, and expression of CTGF (connective tissue growth factor; a.k.a. CCN2) through LPAR1 and LPAR3 in MC3T3-E1 pre-osteoblast cells [170,171]. *Lpar1* null (*Lpar1^−/−^*) mice exhibit dwarfism, short limbs, rib cage deformity, short snout, osteoporotic bones, and low bone mineral density due to decreased osteoblatogenesis and osteoblast differentiation [172]. These results suggest that phospholipids and their receptors positively regulate bone formation [173,174].

**Figure 4:**
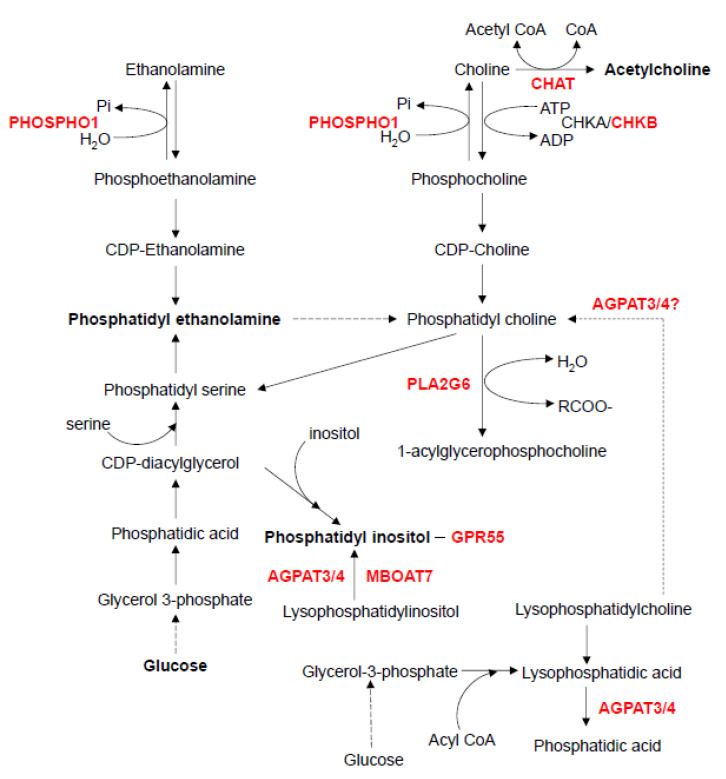
Phospholipid metabolism. This figure was drawn based on the ethanolamine and choline metabolism pathway map from the KEGG website. AGPAT3, 1-Acylglycerol-3-phosphate O-acyltransferase 3; AGPAT4, 1-Acylglycerol-3-phosphate O-acyltransferase 4; CHAT, choline O-acetyltransferase; CHKA, choline kinase alpha; CHKB, choline kinase beta; GPR55, G protein-coupled receptor 55; MBOAT7, membrane bound O-acyltransferase domain containing 7; PHOSPHO1, phosphoethanolamine/phosphocholine phosphatase 1; PLA2G6, phospholipase A2 group VI.

In the phospholipid metabolic pathway, mice with a deficiency of either choline O-acetyltransferase (*Chat*), choline kinase beta (*Chkb*), phosphoethanolamine/phosphocholine phosphatase (*Phospho1*), phospholipase A2 group VI (*Pla2g6*), membrane-bound O-acyltransferase domain containing 7 (*Mboat7*)*,* 1-acylglycerol-3-phosphate O-acyltransferase 3 (*Agpat3*), or 1-acylglycerol-3-phosphate O-acyltransferase 4 (*Agpat4*) exhibit defects in bone formation and/or homeostasis (Figure 4). 

ChAT catalyzes the synthesis of acetylcholine from acetyl-CoA and choline. Acetylcholine plays a crucial role in synaptic transmission, particularly at neuromuscular junctions, in the regulation of skeletal muscle activity. *Chat^−/−^* mice display kyphosis at birth due to impaired skeletal muscle development and dysfunctional neurotransmission within muscles [175,176,177].

Choline kinase [a.k.a. ethanolamine kinase (EK)] includes two isozymes, CHKA and CHKB, and catalyzes the first step of the biosynthesis of phosphatidylcholine and phosphatidylethanolamine. Mice with a 1.6-kb intragenic deletion within the choline kinase beta (*Chkb*) gene (*Chkb^rmd/rmd^* mice) exhibit severe dystrophy in hindlimb skeletal muscles and bone deformity in the forelimbs due to chondrocyte differentiation arrest and suppression of cartilage matrix degradation in the growth plates [178,179]. Moreover, mice with an A-to-T transversion at the start codon of the *Chkb* gene (*Chkb^flp/flp^* mice) exhibit deformity in the forelimbs and low bone mass via increased osteoclast number, accelerated bone matrix resorption by osteoclasts, and reduced mineralization activity in osteoblasts [180].

In the next step of the phospholipid metabolic pathway, PHOSPHO1, a membrane-bounded matrix vesicle phosphatase, catalyzes the dephosphorylation of both phosphoethanolamine and phosphocholine, thus producing Pi, and also plays a role in the initiation of the calcification process, particularly in skeletal tissues [181,182]. *Phospho1^−/−^* mice display growth plate abnormalities, low bone mineral density, fragile bones, osteomalacia, and thoracic scoliosis due to decreased mineralization during endochondral ossification [183,184], and also exhibit hypomineralized dentin and enamel in the teeth [185,186].

Another enzyme that participates in phospholipid metabolism is PLA2G6, which breaks down phosphatidylcholine to produce arachidonic acid and lysophospholipid [187]. The absence of *Pla2g6* in mice results in neurodegeneration and kyphosis due to motor dysfunction, without any bone defect, as seen in patients with mutations in this gene [188]. 

MBOAT7, a member of the acyltransferase family, catalyzes the conversion of lysophosphatidylinositol (LPI) into phosphatidylinositol; *Mboat7*-deficient mice (*Mboat7^−/−^* mice) display domed-shaped calvaria [189]. Since LPI bound to G-protein coupled receptor 55 (GPR55) and dysregulated LPI‒GPR55 signaling results in metabolic diseases [190], treatment of monocytes and pre-osteoclasts with either LPI or a GPR55 agonist, O-1602, induces osteoclastogenesis through osteoclast differentiation/polarization and bone resorption activity in vitro [191]. Moreover, *Gpr55^−/−^* mice show an osteopetrosis-like phenotype, a reduced number of osteoclasts, and increased bone mass, without effects on osteoblastogenesis and bone matrix formation [191].

AGPAT3 and its paralog AGPAT4 are other members of the acyltransferase family of proteins; they convert lysophosphatidic acid (LPA) to phosphatidic acid during phospholipid biosynthesis. An increasing number of studies indicate that LPA differentially affects various bone cells via LPA receptors. For example, LPA accelerates osteogenic differentiation of bone marrow mesenchymal stromal cells, stimulates osteoblast proliferation and maturation, and promotes bone resorption by osteoclasts [192]. Accordingly, *Agpat3*^+*/−*^ and *Agpat4^−/−^* mice show decreased bone mineral content, although the amount of LPA may be increased (reported by the IMPC).

## 8. Conclusions

There are a variety of metabolic pathways that may affect bone development and homeostasis. In recent years, it has been reported that imbalance of cellular and systemic metabolism is associated with various bone diseases and developmental defects [193,194,195,196]. Several of these metabolic pathways have been extensively examined with great interest in clinical cohort studies and mouse genetic studies. While there is significant evidence showing a possible link between bone diseases and metabolic disorders, the specific players and molecular interactions in these metabolic networks remain to be determined. In this review, we focused on recent discoveries concerning metabolic pathways potentially associated with bone diseases. As indicated by several reports, the nutritional and pharmacological manipulation of these pathways may prevent or improve bone diseases caused by metabolic dysfunction.

## Figures and Tables

**Table 1:** Role of the metabolic pathways in bone cells.

Metabolic Pathway	Bone Cells	Cascade/Enzyme/Molecule	Function
Cholesterol synthesis	Osteoblasts	Farnesylation	To induce osteoblast differentiation and mineralization
		Geranylgeranylation	To suppress osteoblast differentiation and mineralization
		Deficiency for the farnesyl-diphosphate farnesyltransferase (*FDFT1*) gene Farnesyl pyrophosphate (FPP)	To suppress osteoblast differentiation and mineralization
		Deficiency for the 7-dehydrocholesterol reductase (*DHCR7*) gene	To accelerate osteoblast differentiation and mineralization via ciliogenesis and accelerate bone formation
		Deficiency for the insulin-induced gene 1 and 2 (*INSIG1* and *INSIG2*) genes	To suppress osteoblast differentiation and mineralization via ciliogenesis and suppress bone formation
	Osteoclasts	Geranylgeranylation	To induce osteoclast differentiation
	Chondrocytes	Deficiency for *INSIG1* and *INSIG2* Deficiency for the sterol regulatory element-binding protein cleavage-activating protein (*SCAP*) gene	To suppress chondrocyte differentiation
		*DHCR7* deficiency	To suppress chondrocyte proliferation
		Deficiency for the transmembrane 7 superfamily member 2 (*TM7FS2*) gene Lamin B deficiency	To disorganize the growth plate
	Mesenchymal stem cells	Farnesylation	To induce osteoblast differentiation and mineralization
		Geranylgeranylation	To suppress osteoblast differentiation and mineralization
Fatty acid metabolism	Osteoblasts	Long-chain fatty acids Essential fatty acids	To induce osteoblast differentiation
		Deficiency for the carnitine palmitoyltransferase 2 (CPT2)	To suppress osteoblast differentiation
	Osteoclasts	Long-chain fatty acids Short-chain fatty acids	To suppress osteoclast differentiation
Glycolysis & Gluconeogenesis	Osteoblasts	Glucose	To maintain ossification and osteoblast differentiation
		Deficiency for the NAD-dependent malic enzyme, mitochondria (*ME2*) gene	To suppress proliferation and osteoblast differentiation
		Deficiency for the glucose 6-phosphatase (*G6Pase*) gene	To suppress ossification
		Deficiency for the pyruvate dehydrogenase kinase 4 (*PDK4*) gene [activation of pyruvate dehydrogenase complex (PDC)]	To fail to induce osteoclastogenesis
	Osteoclasts	Glucose	To maintain osteoclastogenesis
		*PDK4* deficiency (activation of PDC)	To enhance osteoclastogenesis and bone resorption
		Deficiency for the pyruvate dehydrogenase kinase 2 (*PDK2*) gene (activation of PDC)	To suppress osteoclastogenesis and bone resorption
	Chondrocytes	Glucose	To maintain growth plate development
	Bone marrow mesenchymal stem cells	*PDK4* deficiency (activation of PDC)	To suppress osteoclastogenesis
Glycogenolysis & Glycogenesis	Osteoblasts	Deficiency for the ectonucleotide pyrophosphatase/phosphodiesterase 1 (*ENPP1*) gene	To accelerate mineralization
TCA cycle	Osteoblasts	D-2-hydroxyglutarate	To suppress bone formation
	Chondrocytes	Oncogenic point mutations in the isocitrate dehydrogenase 1, cytosolic (IDH1)	To enhance chondrocyte proliferation and differentiation
	Mesenchymal stem cells	D-2-hydroxyglutarate	To suppress osteoblast differentiation
Phospholipid metabolism	Osteoblasts	Phosphatidylserine	To enhance osteoblast differentiation
		Lysophosphatidic acid	To enhance osteoblast differentiation and mineralization
	Osteoclasts	Phosphatidylserine	To maintain osteoclast fusion and apoptosis
		lysophosphatidylinositol (LPI) and G protein-coupled receptor 55 (GPR55) agonists	To enhance osteoclastogenesis, osteoclast maturation, and bone resorption

**Table 2:** Skeletal phenotype in mice with metabolic aberrations.

Metabolic Pathway	Mutant Mice	Skeletal Phenotype
Cholesterol synthesis	*Cyp51^−/−^*	cleft palate, micrognathia, brachycephaly, microglossia/aglossia, polydactyly/syndactyly, and malformation of long bones
	*Dhcr7^−/−^*	cleft palate, postaxial polydactyly, 2-3 toe syndactyly, microcephaly, and micrognathia
	*Insig1^−/−^;Insig2^−/−^*	midline cleft face or cleft palate, and micrognathia
	*Wnt1-Cre;Insig1^F/F^;Insig2^−/−^*	osteogenesis imperfecta-like calvaria
	*Prrx1-Cre;Insig1^F/F^;Insig2^−/−^*	disorganized growth plate, short limbs, and dwarfism
	*Col2a1-Cre;Insig1^F/F^;Insig2^−/−^*	disorganized growth plate, short limbs, midline cleft, and dwarfism
	*Lbr^−/−^*	disorganized hypertrophic chondrocytes, immature trabecular bone, and growth retardation
	*Lbrβ* ^+*/−*^ *; Tm7sf2^∆4-7/∆4-7^*	short growth plates, less trabecular bones, growth retardation, and cleft palate
	*Nsdhl*^+*/−*^ female	skeletal dysplasia and chondrodysplasia punctata
	*Sc5d^−/−^*	cleft palate, micrognathia, tooth agenesis of lower incisors, calvaria hypomineralization, malformation of long bones, and syndactyly/polydactyly
	*Prrx1-Cre;Scap^F/F^* *Col2a1-Cre;Scap^F/F^*	disorganized growth plate, short limbs, and dwarfism
Fatty acid metabolism	*Acsbg2^−/−^*	low bone mineral density
	*Cd36^−/−^*	osteoblast differentiation defect and lower bone mass in adults
	*Gpr40^−/−^*	osteoporosis
	*Cpt2^−/−^*	abnormal vertebrae morphology
	*Oc-Cre;Cpt2^F/F^* (osteocalcin-Cre)	decreased bone acquisition
	*Ppt1^−/−^*	thick calvaria
Glycolysis & Gluconeogenesis	*Aldob^−/−^*	low bone mineral content and density
	*G6pc^−/−^*	cartilage dysplasia
	*Pgd^+/−^*	low bone mineral content and density
	*Dermo1-Cre;Slc2a1^F/F^*	bone mineralization defects in endochondral and intramembranous ossification
	*Lys2-Cre;Slc2a1^F/F^* female	osteoclastogenesis defects and increased trabecular bone mass
	*Prx1-Cre;Slc2a1^F/F^*	disorganized growth plate, suppression of cartilage matrix synthesis, and short long bones
	*Slc37a4^−/−^*	delayed bone development
Glycogenolysis & Glycogenesis	*Enpp1^−/−^twy* (spontaneous) *Enpp1 ^c.T737A^*	bone mineralization defects
TCA cycle	*Col2a1-Cre;Idh1^R132Q/+^*	growth plate disorganization and cartilaginous dysplasia
	*Sdhc^+/−^* female	low bone mineral contents
Phospholipid metabolism	*Agpat3^+/−^* *Agpat4^−/−^*	low bone mineral contents
	*Chkb^rmd/rmd^*	chondrocyte differentiation arrest, suppression of cartilage matrix degradation, and bone deformity in the forelimbs
	*Chkb^flp/flp^*	deformity in the forelimbs and low bone mass
	*Gpr55^−/−^*	osteopetrosis-like bone
	*Lpar1^-/^*	dwarfism, short limbs, rib cage deformity, short snout, osteoporotic bones, and low bone mineral density
	*Mboat7^−/−^*	domed shape calvaria
	*Phospho1^−/−^*	growth plate differentiation defects, low bone mineral density, fragile bones, osteomalacia, and hypomineralized amelogenesis imperfecta

## Abbreviations

**6PGD**, 6-phosphogluconate dehydrogenase; **7****-DHC**, 7-dehydrocholesterol; ***Acadl1***, acyl-CoA dehydrogenase, long chain; ***Acsl4***, acyl-CoA synthetase long-chain family member 4; ***Acsl5***, acyl-CoA synthetase long-chain family member 5; ***Acsbg2***, acyl-CoA synthetase bubblegum family member 2; ***Agl***, amylo-alpha-1, 6-glucosidase, 4-alpha-glucanotransferase; ***Agpat3***, 1-acylglycerol-3-phosphate O-acyltransferase 3; ***Agpat4***, 1-acylglycerol-3-phosphate O-acyltransferase 4; ***Aldob***, aldolase, fructose-bisphosphate B; **ATP**, adenosine triphosphate; ***Bai1***, brain-specific angiogenesis inhibitor 1; **BMD**, bone mineral density; **CD36**, CD 36 molecule; **c-FOS**, FBJ osteosarcoma oncogene; ***Chat***, choline O-acetyltransferase; **CHILD**, Ichthyosiform nevus and Limb Defects; ***Chka***, choline kinase alpha; ***Chkb***, choline kinase beta; ***Col10a1***, collagen type X alpha 1 chain; **COPII**, coat protein complex II; ***Cpt2***, carnitine palmitoyltransferase 2; **CREB**, cAMP response element-binding protein; ***Ctgf***, connective tissue growth factor; ***Cyp51***, cytochrome P450, family 51; **D-2HG**, D-2-hydroxyglutarate; **DAG**, diacylglycerol; ***Dhh***, desert hedgehog; ***Dhcr7***, 7-dehydrocholesterol reductase; ***Dhcr24***, 24-dehydrocholesterol reductase; ***Ennp1***, ectonucleotide pyrophosphatase/phosphodiesterase 1; **ENU**, N-ethyl-N-nitrosourea; **ER**, endoplasmic reticulum; **ERK1 (MAPK3)**, mitogen-activated protein kinase 3; **ERK2 (MAPK1)**, mitogen-activated protein kinase 1; **ERRα**, estrogen related receptor alpha; **F6P**, fructose-6-phosphate; **FAT**, fatty acid translocase; ***Fdft1***, farnesyl-diphosphate farnesyltransferase 1; **FDPS**, farnesyl diphosphate synthase; ***Fgfr3***, fibroblast growth factor receptor type III; **FPP**, farnesyl pyrophosphate; **G1P**, glucose 1-phosphate; **G6P**, D-glucose-6-phosphate; ***G6pase***, glucose 6-phosphatase; ***G6pc***, glucose-6-phosphatase catalytic subunit; ***Gad1***, glutamate decarboxylase 1; ***Gfpt1***, glutamine fructose-6-phosphate transaminase 1; ***Gfpt2***, glutamine fructose-6-phosphate transaminase 2; **GGPP**, geranylgeranyl diphosphate; **GLUT1**, glucose transporter 1; ***Gpr40***, G protein-coupled receptor 40; ***Gpr41***, G protein-coupled receptor 41; ***Gpr43***, G protein-coupled receptor 43; ***Gpr55***, G protein-coupled receptor 55; ***Gpr120***, G protein-coupled receptor 120; **GGPPS1**, geranylgeranyl diphosphate synthase; **GH**, growth hormone; ***Gpt1***, glutamic pyruvic transaminase 1; **GSD-Ia**, glycogen storage disease type Ia; **GSD-Ib**, glycogen storage disease type Ib; **GSD-III**, glycogen storage disease type III; **GTP**, guanosine triphosphate; **HDL-C**, high-density lipoprotein cholesterol; **HMG-CoA**, 3-hydroxy-3-methylglutaryl-coenzyme A; **HMGCR**, HMG-CoA reductase; ***Hsd17b7***, hydroxysteroid (17-beta) dehydrogenase 7; ***Hsd17b12***, hydroxysteroid (17-beta) dehydrogenase 12; ***Idh1***, isocitrate dehydrogenase 1, cytosolic; **IGF1**, insulin-like growth factor 1; **IGF1R**, insulin-like growth factor 1 receptor; ***Ihh***, Indian hedgehog; ***Insig1***, insulin-induced gene; ***Insig2***, insulin-induced gene 2; **LPA**, lysophosphatidic acid; ***Lpar1***, lysophosphatidic acid receptor 1; ***Lpar3***, lysophosphatidic acid receptor 3; **LPI**, lysophosphatidylinositol; **LRB**, lamin B receptor; ***Mboat7***, membrane bound O-acyltransferase domain containing 7; ***Mcat***, malonyl-CoA-acyl carrier protein transacylase; ***Me2***, NAD-dependent malic enzyme, mitochondria; **NADH**, nicotinamide adenine dinucleotide; **NADP/NADPH**, nicotinamide adenine dinucleotide phosphate; **N-BP**, nitrogen containing bisphosphonate; **NFATc1**, nuclear factor of activated T cells 1; **NF****κB**, nuclear factor kappa-light-chain-enhancer of activated B cell; ***Nsdhl***, NAD(P) dependent steroid dehydrogenase-like; **PA**, phosphatidic acid; **Pi**, phosphate; **PDC**, pyruvate dehydrogenase complex; ***Pdk2***, pyruvate dehydrogenase lipoamide kinase isozyme 2; ***Pdk4***, pyruvate dehydrogenase kinase 4; ***Pgd***, phosphogluconate dehydrogenase; ***Phospho1***, phosphoethanolamine/phosphocholine phosphatase; **PIP2**, phosphatidylinositol-(4,5)-bisphosphate; ***Pla2g6***, phospholipase A2 group VI; **POR**, cytochrome P450 oxidoreductase; **PPi**, pyrophosphate; ***Ppt1***, palmitoyl-protein thioesterase 1; **RANKL (TNFS11)**, receptor activator of nuclear factor kappa-B ligand (tumor necrosis factor (ligand) superfamily, member 11); ***Runx2***, runt related transcription factor 2; ***Sc4mol***, methylsterol monooxygenase 1; ***Sc5d***, sterol-C5-desaturase; ***Scap***, sterol regulatory element-binding protein cleavage-activating protein; **SDH**, succinate dehydrogenase; ***Sdhc***, succinate dehydrogenase complex subunit C; ***Shh**,* sonic hedgehog; ***Slc2a1***, solute carrier family 2 member 1; **SLOS**, Smith-Lemli-Opitz syndrome; ***Slc37a4***, solute carrier family 37 member 4; **SMO**, smoothened; **SNP**, single nucleotide polymorphism; ***Srebp***, sterol regulatory element-binding protein; ***Stab2***, stabilin-2; **TCA cycle**, tricarboxylic acid cycle, *aka* citric acid cycle or Krebs cycle; **TG**, triglyceride; ***Tim4 (Timd4)***, T-cell membrane protein 4 (T-cell immunoglobulin and mucin domain containing 4); ***Tm7sf2***, transmembrane 7 superfamily member 2; **UDP**, uridine diphosphate; **UGP2**, UDP-glucose pyrophosphorylase 2; **UTP**, uridine triphosphate.
